# Blocking Tumoral Angiogenesis VEGF/VEGFR Pathway: Bevacizumab—20 Years of Therapeutic Success and Controversy

**DOI:** 10.3390/cancers17071126

**Published:** 2025-03-27

**Authors:** Elena Chitoran, Vlad Rotaru, Daniela-Cristina Stefan, Giuseppe Gullo, Laurentiu Simion

**Affiliations:** 1 Medicine School, “Carol Davila” University of Medicine and Pharmacy, 050474 Bucharest, Romania; 2 General Surgery and Surgical Oncology Department I, Bucharest Institute of Oncology “Prof. Dr. Al. Trestioreanu”, 022328 Bucharest, Romania; 3 Department of Obstetrics and Gynecology, Villa Sofia Cervello Hospital, University of Palermo, 90146 Palermo, Italy

**Keywords:** angiogenesis, angiogenesis inhibitors, VEGF, VEGF receptors, Bevacizumab, molecular mechanisms, resistance to antiangiogenetic drugs

## Abstract

The “angiogenesis switch”—defined as the active process by which solid tumors develop their own circulation—plays an important role in both tumoral growth and propagation. As the malignant tumor grows and reaches a critical size, the metabolic needs as a function of an ever-increasing distance to the nearest emergent blood vessel, can no longer be covered by the microenvironment of the peritumoral tissue. Although a relatively discrete process, the “angiogenic switch” acts as a limiting stage of tumoral development present from the avascular hyperplasia phase to the vascularized neoplastic phase, providing support for tumor expansion and metastasis. Over time, research has focused on blocking the angiogenetic pathways (such as VEGF/VEGFR signaling axis) leading to the development of targeted therapeutic agents such as Bevacizumab. Objectives: We conducted a review of the molecular principles of tumoral angiogenesis and we tried to follow the history of Bevacizumab from its first approval for human usage 20 years ago to current days, focusing on the impact this agent had in solid tumor therapy. A comprehensive review of clinical trials pertaining to Bevacizumab (from the era of the preclinic trials leading to approval for human usage, to the more recent randomized trial focusing on combination targeted therapy) further details the role of this drug. We aimed to establish if this ancient drug continues to have a place in modern oncology. Conclusions: Bevacizumab, one of the first drugs targeting tumoral microenvironment, remains one of the most important oncologic agents blocking the VEGF/VEGFR angiogenic pathway. otherwise, history of 20 years marked by numerous controversies (ranging from methodological errors of clinical trials to withdrawal of approval for human usage in breast cancer patients, from discussions about severe side effects to resistance to therapy and limited efficacity), Bevacizumab continues to provide an optimal therapeutic option for many solid tumors that previously had little to no means of treatment, improving otherwise bleak outcomes. Even in the era of personalized precision oncology, Bevacizumab continues to be a key element in many therapeutic regimens both as monotherapy and in combination with newer targeted agents.

## Simple Summary

Tumoral angiogenesis plays a central role in the development and progression of solid tumors and VEGF/VEGFR pathway is the principal signaling axis controlling this process. Bevacizumab, the first therapeutic agent inhibiting tumoral angiogenesis by blocking VEGF/VEGFR pathway, is a drug with a long and controversial history but which remains as current as when first approved for clinical use. We discuss the tumoral angiogenesis and the role Bevacizumab plays in cancer therapy—does this ancient drug still have a place in modern oncology?

## 1. Introduction

The “angiogenesis switch”—defined as the active process by which solid tumors through internal means develop their own circulation [1,2,3]—plays an important role in both tumoral growth and propagation. As the malignant tumor grows and reaches a critical size, the metabolic needs (both nutritive and eliminatory), as a function of an ever-increasing distance to the nearest emergent blood vessel, can no longer be covered by the microenvironment of the peritumoral tissue. Although a relatively discrete process, the “angiogenic switch” acts as a limiting stage of tumoral development, present from the avascular hyperplasia phase to the vascularized neoplastic phase and providing support for tumor expansion and metastasis [1,2,4]. Moreover, experimental models showed that the progression of tumors beyond 0.2–2 mm is highly dependent on neo-angiogenesis [5].

The first observations on the increased vasculature of tumors appeared in German literature in the late 1800s and belongs to Rudolph Virchow. In 1927, Warren Lewis observes that the architecture of tumoral vascularization depends on cancer type and proposes a correlation between tumoral microenvironment factors and the growth and morphological characteristics of tumor vessels [6]. However, this theory was not very widely acknowledged by his peers, which pointed out that many aggressive tumors had an apparent poor blood supply. Although this effect was confirmed—the aggressive tumors often having a rapid growth pattern resulting in the inability of generating new blood vessels to compensate increased needs and subsequent central necrosis which can be observed in post-mortem examination—it did not provide a sufficient argument against tumoral angiogenesis. In 1939, Gordon et al. used a previously described technique (Sandison, 1928) for investigating the potential correlation between increasing vascular supply and tumor growth, observing that the transplantation of tumors triggers an intense angiogenic response in test animals. These findings lead them to postulate, for the first time, the release of some sort of tumor-dependent vascular growth factor [7]. In 1945, Glenn Algire and his colleagues published a study furthering Gordon’s theory, by introducing a quantitative approach to assess blood-vessel growth (daily counts of blood vessels and subsequent comparison to the observed tumor size). They observed that transplanted tumors featured a marked increased vascularity, which did not occur in surrounding normal tissues, and which, interestingly, preceded the rapid growth process of tumors [8]. These findings led Algire to conclude that the rapid tumoral growth phase is dependent on tumoral cell’s ability to induce new vessel growth, one of the most crucial steps in tumorigenesis. Moreover, this ability gives tumoral cells a selective in vivo advantage over surrounding normal cells.

It was not until the late 1960s that the first evidence of angiogenic factors released by tumoral cells was available. Two separate collectives (Greenblatt and Shubik from Chicago Medical School and Ehrmann and Knoth at Harvard Medical School [9,10]) showed that transplanted melanoma cells and, respectively, choriocarcinoma cells promote blood vessel proliferation even when a filter is interposed between the malignant cells and the tissues of the host. Although they could not entirely rule out the possibility of direct cell-to-cell contact between malignant and normal cells through the filter pores, thus causing the induction of neo-angiogenesis, they stated that a more plausible explanation for this phenomenon was tumoral cells releasing a stimulating diffusible factor.

In the context of these previous studies, the postulates of Judah Folkman, who suggested for the first time in 1971, that tumor angiogenesis can be a potential therapeutic target [3], were the logical conclusion and led to the orientation of cancer research, away from classical cell-centered therapies to therapeutic modalities aimed at inhibiting pathological angiogenesis. Thus, a new field of oncology was born [3,11,12,13,14] and blocking the VEGF/VEGFR signaling pathway became the main target in the development of new therapeutic agents with antiangiogenic effect for solid tumors, among which Bevacizumab remains the oldest and the most iconic.

## 2. Mechanisms of Tumoral Angiogenesis

During the “angiogenetic switch”, the tumoral cells form an integrated system with cells composing the tumor stroma (like tumor-associated fibroblasts, pericytes, inflammatory, and endothelial cells) and together they orchestrate a complex multifactorial reaction culminating in new vessels production.

**Figure 1:**
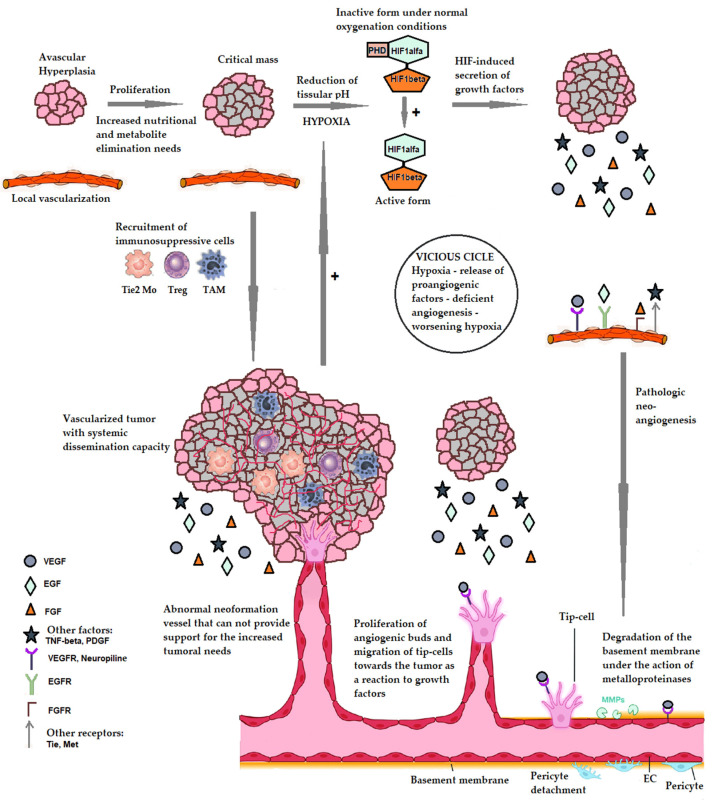
The mechanism of pathological tumoral angiogenesis. PHD—prolyl hydroxylase—an enzyme that under normal oxygenation conditions causes the hydroxylation of the alpha subunit of HIF-1 resulting in its inactivation. HIF1alpha and HIF1beta—the alpha and beta subunits of HIF-1. EC—endothelial cells. VEGF—vascular endothelial growth factor. EGF—epithelial growth factor. FGF—fibroblast growth factor. TNF-alpha—tumor necrosis factor alpha. PDGF—Platelet-derived growth factor. VEGFR—receptor for VEGF. EGFR—EGF receptor. FGFR—FGF receptor. Tie, Met—tyrosine-kinase receptors with a role in tumor proliferation. TAM—tumor associated macrophage. Treg—T cells with regulatory role. Tie2 Mo—monocytes expressing the Tie2 receptor for angiopoietin. References pertaining to the making of the figure above: [1,2,3,4,16,20,21,22,23,24,25,26,27,28,29,30,31,32,33,34,35].

The proliferation of the tumoral cell mass determines the increased need for nutritional intake and the elimination of metabolic products that exceed the capacity of the peritumoral tissues, leading to changes in tissular pH and hypoxia phenomena. Hypoxia is the most important trigger of proangiogenic factor production both from the cancer cells and from the stromal fibroblasts and macrophages. The subsequent growth of solid tumors is dependent on the network of new vessels developed under the influence of HIF-1 (Hypoxia-inducible factor 1). HIF-1 in hypoxia conditions passes from an inactive form into an active form that determines the secretion of growth factors by the tumoral cells. HIF-1 also induces the release of pro-angiogenetic factors from the immunosuppressive cells recruited during the inflammatory processes in the peritumoral tissues. Under the influence of the released growth factors, endothelial cells with proliferative potential (called “Tip-cells”) are recruited, and will initiate the appearance of angiogenic buds in local vessels. The “budding” process is aided by the degradation of the endothelial basement membrane under the action of metalloproteinases (MMPs) and the detachment of pericytes. The resulting neoformation vessels are, however, aberrant, hyper-permeable, and collapsable under the weight of the growing tumoral mass, and they cannot meet the growing needs of the tumor. As a result, the aggravation of local hypoxia occurs, resulting in a vicious cycle: hypoxia—the release of proangiogenic factors—deficient neo-angiogenesis—aggravation of local hypoxia. This vicious cycle is the most important factor differentiating tumoral angiogenesis from the physiological version (the latter being a self-limiting process where this vicious cycle does not occur). This mechanism is detailed in Figure 1.

**Figure 2:**
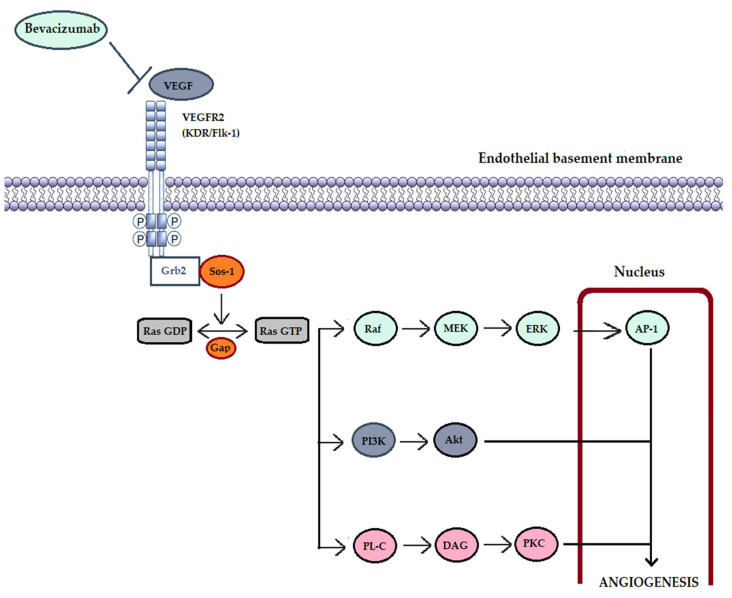
VEGF-dependent intracellular mechanisms involved in angiogenesis. VEGF (vascular endothelial growth factor); VEGFR2 (VEGF receptor 2); Grb2 (Growth factor receptor-bound protein 2)—is an adapter protein involved in signal transduction and cellular communication; Sos-1 (son of sevenless-1)—regulatory protein (activator) of Ras proteins by promoting the transformation of GDP (guanosine-diphosphate) into GTP (guanosine-triphosphate); GAP—GTP-ase-activating protein; Raf (Raf protein kinase)—key intermediary role between membrane GTPases and downstream kinases (MEK and ERK); AP-1 (activating protein-1)—is a transcription factor involved in differentiation, proliferation, cell survival and apoptosis; PI3K (phosphatidylinositol 3-kinase) together with Akt (Ak strain transforming) form an intracellular signaling pathway involved in cell metabolism, growth, proliferation and cell survival; PL-C (phospholipase C); DAG (diacylglycerol); PKC (protein kinase C)—kinase involved in the modulation of multiple regulatory molecules of the cell cycle.

Angiogenesis in tumors is a highly regulated biological process, with multiple factors playing a role. Among the regulating factors, VEGF (vascular endothelial growth factor) is the most important and well known. VEGF is a positive regulation factor for angiogenesis through a mechanism involving the activation of tyrosine-type receptors kinase (TRK) and co-receptors (neuropilines 1 and 2) through autophosphorylation. Thus, VEGF triggers several intracellular signaling pathways involved both in angiogenesis and inflammation (these mechanisms are detailed in Figure 2). The expression of VEGF is also regulated through multiple cytokines (like IL-1, IL-6, and nitric oxide), growth factors (epidermal growth factor—EGF, platelet derived growth factor—PDGF or tumor necrosis factor alpha—TNF-α,), and hormones (such as thyrotropin, steroid hormones, insulin, and corticotropin) [15]. VEGF expression is also stimulated by the loss of tumor suppressor genes (like p53) and the activation of various oncogenes (such as ras, v-src, and Her2). But the most intense factor stimulating the overexpression of VEGF is still hypoxia [16], which triggers the stabilization of the alpha subunit of HIF-1. The alpha subunit of HIF-1 is unstable under normal oxygenation conditions and becomes stable under hypoxia conditions. Hypoxia also acts as a stabilization agent for VEGF messenger RNA. The overexpression of VEGF is highly correlated with metastatic dissemination and lymphatic involvement. From a clinical point of view, patients with higher VEGF levels have worse survival rates than those with low or negative levels. Also, preoperative levels of VEGF are correlated with more advanced cancerous disease [17,18]. It was postulated that levels of VEGF may predict the metastatic potential and is an independent risk factor to nodal status and adjuvant chemotherapy [19].

It becomes apparent as to why, over time, research focused on blocking the VEGF/VEGFR signaling pathway that became the main target in the development of new antiangiogenic drugs, resulting in the development and approval of numerous therapeutic agents. However, anti-neoplastic therapies focused exclusively on the inhibition of the appearance/growth of neoplastic vessels and/or the destruction of pre-existing vessels, remain suboptimal showing limited clinical efficiency [24,36,37], severe adverse reactions, and resistance to therapy.

## 3. Bevacizumab—One of the Most Relevant Anti-Angiogenetic Drugs in Solid Tumors

After VEGF (formerly called vascular permeability factor [38]) was shown to be the dominant pro-angiogenic factor in many solid tumors [39], the search for the best strategy to block the VEGF–VEGFR axis [40,41] began. In 1992, Kim and colleagues generated four types of murine monoclonal antibodies (mAbs) (A3.13.1, A4.6.1, B2.6.2, and B4.3.1) using three types of recombinant human VEGF (VEGF-A121, VEGF-A165, and VEGF-A189) as immunogens. These antibodies belong to the IgG1 immunoglobulins and recognize two epitopes of the immunogen. The monoclonal antibody B2.6.2 recognizes a discontinuous epitope of recombinant human VEGF and binds only to VEGF-A165 and VEGF-A189, while mAb A4.6.1 recognizes a continuous epitope and shows high affinity for all types of immunogens (VEGF-A121, VEGF-A165, and VEGF-A189). Subsequent tests (in vivo vascular permeability test, in vivo embryonic chicken angiogenesis assay, and in vitro bovine adrenal cortex endothelial cell proliferation assay) showed that mAb4.6.1 has the highest VEGF neutralizing activity [42]. The use of murine antibodies for therapeutic applications in human subjects is, however, limited due to immune response and toxicity. To overcome these shortcomings, the A4.6.1 antibody was humanized. The first attempt at humanization dates back to 1997 and was made by Presta et al. [43]. The humanized version of A4.6.1 contained the amino acid sequences of six complementarity determining regions (CDRs) of the original antibody grafted onto a human IgG1 consensus framework consisting of heavy (H-heavy) and light (L-light) chains, containing variable and constant regions. The resulting antibody, however, had a significantly weaker affinity for VEGF than the parent antibody. Genentech, Inc., South San Francisco, continued the humanization process of mAb A4.6.1, changing seven residues of the variable components of the heavy chains and one of the variable components of the light chains from human to murine, restoring VEGF binding equivalence to values similar to those of the parent antibody [42,43]. This resulted in a recombinant humanized monoclonal antibody that neutralizes all forms of VEGF-A similar to A.4.6.1. This is known as Bevacizumab which was first approved and marketed under the trade name Avastin^®^.

And so, the history of more than 20 years of Bevacizumab began, the drug providing an effective therapeutic option for a number of advanced solid tumors that were previously known to have a poor prognosis. Although other antiangiogenic agents blocking VEGF angiogenic pathway and/or other pro-angiogenic signaling pathways have been developed, Bevacizumab remains the most widely used and most thoroughly characterized angiogenesis inhibitor [44,45]. Close to 40,000 patients have received Bevacizumab in various clinical trials and more than 3.5 million patients were treated as part of the prescribed oncological regimen [46].

## 4. Efficacy and Safety of Bevacizumab in Solid Tumors

To better highlight the efficacity and safety of Bevacizumab in various solid tumors, we will discuss these aspects for each neoplastic disease, for which Bevacizumab has gained approval for human usage. The results of the main studies available are summarized in Table 1.

### 4.1. Colorectal Cancer

Bevacizumab (Avastin^®^) was approved in February 2004 in the United States as a first-line treatment for metastatic colorectal cancer, in combination with standard chemotherapy. Thus, it became the first angiogenesis inhibitor approved by the FDA (Food and Drug Administration) for human use [47]. In January 2005, it also received approval from the EMA (European Medicines Agency). In June 2006, the FDA also approved its use in combination with 5-FU-based chemotherapy as a second-line treatment for metastatic colorectal cancer [48]. The clinical efficacy of Bevacizumab in metastatic colorectal cancer was evaluated in the pivotal study AVF2107g and the supporting study AVF0780g. The activity of Bevacizumab monotherapy in colorectal cancer was analyzed in the E3200 study.

**Table 1:** Results of main studies leading to Bevacizumab approval for use in human cancer patients for the most important indications.

Study Name	Study Type	Cohort	Intervention	Outcomes/Endpoints	Main Results
**Colorectal cancer**					
AVF2107g [49,50,51]	Phase III, multicenter, active-controlled study, sponsored by Genentech, first-line therapy	813 patients in 163 centers, mCRC	Arm I: 411 patients—IFL for a total of 4 weeks of each 6 week-cycle (Saltz regimen) + PL.	Primary endpoint: OS Secondary endpoints: PFS, RR, duration of response, QoL and patient safety.	Adding Bev to IFL improved OS (20.3 months in arm II vs. 15.6 months in arm I, *p* < 0.0001). Adding Bev to IFL also improved PFS (10.6 vs. 6.2 months, *p* < 0.0001), RR (34.6% in arm I vs. 44.5% in arm II, *p*-value = 0.0036), and the duration of response (10.35 vs. 7.06 months). Adding Bev did not lead to a more rapid worsening of QoL compared to standard first-line chemotherapy (2.89 vs. 2.73 months).
			Arm II: 402 patients—IFL for a total of 4 weeks of each 6 week-cycle (Saltz regimen) + Bev 5 mg/kg q2w.		
			Arm III *: 110 patients—FL + Bev 5 mg/kg q2w.		
AVF0780g [52,53]	Phase II, randomized, multicenter, multidose study sponsored by Genentech, first-line therapy	104 patients with mCRC	Arm I: 36 patients—FL for a total of 6 weeks of each 8 week-cycle (Roswell Park regimen). Arm II: 35 patients—FL for a total of 6 weeks of each 8 week-cycle (Roswell Park regimen) + Bev 5 mg/kg. Arm III: 33 patients—FL for a total of 6 weeks of each 8 week-cycle (Roswell Park regimen) + Bev 10 mg/kg.	PFS, RR, OS, disease progression.	Disease progression was observed in 26/36 patients in arm I, in 22/35 patients in arm II and in 23/33 patients in arm III. The median PFS in the 3 arms was 5.2, 9 (*p* = 0.005), and 7.2 months, respectively (*p* = 0.236). A statistically significant improvement in PFS was observed in arm II, but no significant was observed for higher Bev doses. OS: 13.6 (arm I), 17.7 (arm II) and 15.2 months (arm III), respectively. No significant differences in OS were observed between the arms, but only a trend towards improvement in arm II.
AVF2192g [51]	Phase II, randomized, double-blind, active-controlled	209 patients with mCRC, patients who were not optimal candidates for 1st line irinotecan treatment	Arm I: 105 patients—FL + PL Arm II: 104 patients—FL + Bev 5 mg/kg q2w.	PFS, RR, OS, duration of response.	Improved PFS in arm II: 9.2 vs. 5.5 months (*p* = 0.0002). Duration of response: 9.2 vs. 6.8 months favoring Bev arm. Non-significant results: improvement in OS in arm II—16.6 vs. 12.9 months (*p* = 0.16); better RR in arm II—26% vs. 15.2% (*p* = 0.055).
ECOG E3200 [54]	Phase III randomized, active-controlled, open-label trial, second-line therapy	829 patients with advanced/metastatic CRC, Bev-naive patients	Arm I: 292 patients—FOLFOX-4 Arm II: 293 patients—FOLFOX-4 + Bev Arm III: Bev in monotherapy.	OS, PFS, RR.	Adding Bev to FOLFOX-4 determined significant improvements in OS (13 vs. 10.8 months, *p* = 0.0012), PFS (7.5 vs. 4.5 months, *p* < 0.0001) and RR (22.2% vs. 8.6%, *p* < 0.0001). No significant benefits were observed for Bev monotherapy compared to standard chemotherapy.
**Lung cancer**					
ECOG E4599 [55]	Phase III, randomized, first-line treatment	878 patients with advanced, recurrent and metastatic NSCLC (stage IIIB or IV)	Arm 1 (444 subjects)—standard chemotherapy carboplatin + paclitaxel Arm 2 (434 subjects)—carboplatin + paclitaxel in combination with Bevacizumab.	OS, PFS, RR.	Adding Bev determined increased OS (12.3 vs. 10.3 months, *p* = 0.003), PFS (6.2 vs. 4.5 months, *p* < 0.001) and better RR (35% vs. 15%, *p* < 0.001). There were 15 deaths associated with Bev therapy, including 5 due to massive pulmonary hemorrhage (increased risk of severe adverse effects). OS in patients with adenocarcinoma histology was twice as high compared to the total patient population suggesting an additional benefit of Bev in this subset of patients.
AVAiL [56]	Phase III, placebo-controlled, first-line therapy	1043 patients with advanced or recurrent NSCLC	Arm 1 (326 subjects): standard chemotherapy (cisplatin + gemcitabine) and PL. Arm 2 (331 subjects): standard chemotherapy + 7.5 mg/kg Bev. Arm 3 (329 subjects): standard chemotherapy + 15 mg/kg Bevacizumab. Bev or PL were administered until disease progression.	OS, PFS.	The final analysis confirmed the efficacy of adding Bevacizumab to the cisplatin + gemcitabine combination chemotherapy, demonstrating a statistically significant PFS benefit in both the 7.5 and 15 mg/kgc groups (*p* = 0.0003 and *p* = 0.0456, respectively). The PFS benefit did not translate into overall survival benefits, with OS being >13 months in all arms. No change in the safety profile was observed in elderly patients (over 65) compared to the entire group.
AVAPERL [57,58]	Phase III, randomized, maintenance	253 Patients with advanced non-squamous NSCLC received first-line Bev+ cisplatin + pemetrexed for 4 cycles. Those achieving response or stable disease were randomized 1:1 to maintenance	Arm 1 (125 patients)—Bev 7.5 mg/kg Arm 2 (128 patients) Bev 7.5 mg/kg plus pemetrexed 500 mg/m^2^ q3w Treatment continued until disease progression or unacceptable toxicity.	PFS, OS.	The combination therapy Bev + permetrex proved superior as maintenance after achieving disease stability with cisplatin/permetrex chemotherapy + Bev (50% reduction in the risk of progression compared with Bev monotherapy (median PFS: 10.2 vs. 6.6 months), but without an OS benefit.
JO25567 [59,60,61]	Phase III, open-label, randomized, multicenter, combination therapy	152 Japanese patients with stage IIIB/IV or postoperative recurrent NSCLC, chemotherapy-naïve, with EGFR mutations	Compared erlotinib + Bev (15 mg/kg every 21 days—75 subjects) combination therapy with erlotinib alone (erlotinib 150 mg once daily—77 subjects).	Risk of disease progression.	The study proved the superiority of the combination therapy (46% reduction in the risk of progression; *p* = 0.015). Based on this study, the erlotinib + Bev combination therapy was approved for the treatment of patients with advanced/metastatic NSCLC harboring EGFR mutations.
IMpower150 [62,63]	Phase III, international, open-label 1:1:1l, randomized trial conducted at 240 study centers in 26 countries, First-line	stage IV or recurrent metastatic non squamous NSCLC (for which they had not previously received chemotherapy	Compared Atezolizumab and Bev + chemotherapy combination to Bev/Atezolizumab + chemotherapy alone.	PFS, OS.	38% reduction in the risk of progression (*p* < 0.001) in the Atezolizumab combination arm. This benefit was observed regardless of EGFR/ALK status These results led to the approval of the combination in the first-line treatment of NSCLC.
**Breast cancer**					
ECOG E2100 [64]	Phase III, Randomized 1:1, open-label	722 patients with locally advanced, recurrent, or metastatic breast cancer who had not previously received Bev	Arm 1: Paclitaxel Arm 2: Paclitaxel + Bev.	OS, PFS, RR.	The addition of Bev to chemotherapy resulted in a 5.5 month increase in median PFS (HR 0.48; 95% CI 0.39–0.61; *p* < 0.0001), but no statistically significant improvement in OS (HR 0.87; 95% CI 0.72–1.05; *p* = 0.137). The RR was higher in the Bev arm compared to control (48.9% vs. 22.2%).
**Ovarian, fallopian tube and primary peritoneal cancer**					
GOG-0218 [65]	Phase III, double-blind, PL-controlled, randomized 1:1:1, first-line therapy	1873 women with stage III or IV primary ovarian, fallopian tube, or peritoneal cancer who had undergone suboptimal cytoreductive surgery	Arm I: platinum-based chemotherapy Arm II: platinum-based chemotherapy + Bev 15 mg/kg. Arm III: platinum-based chemotherapy + Bev followed by Bev maintenance.	PFS, OS.	The addition of Bev to chemotherapy resulted in a statistically significant increase in PFS (from 10.3 to 14.1 months, *p* ≤ 0.001). Although there was no significant difference in OS between the arms, in the subgroup of patients with stage IV disease, combined therapy followed by Bev maintenance led to an improvement in OS (median OS 42.8 vs. 32.6 months).
ICON7 [66]	Phase III, open-label, randomized 1:1, first-line therapy	1528 women diagnosed with stage III or IV primary ovarian, fallopian tube, or peritoneal cancer who underwent cytoreductive surgery	Arm I: carboplatin (AUC 5 or 6) and paclitaxel (175 mg/m^2^), q3w, for 6 cycles. Arm II: carboplatin + paclitaxel and Bev 7.5 mg/kg concurrently q3w for 5 or 6 cycles and continued for 12 additional cycles or until progression of disease.	PFS, OS.	The final published results did not prove a significant benefit in PFS or OS. Only the analysis of the subgroup of high-risk patients demonstrated an improvement in PFS in the Bevacizumab-treated arm (16.0 vs. 10.5 months; *p* = 0.001).
PAOLA-1 [67,68]	Phase III, randomized 2:1, double-blind, PL-controlled	806 patients with newly diagnosed, advanced, high-grade ovarian cancer which responded to first-line platinum–taxanes chemotherapy plus Bev. Patients were eligible regardless of surgical outcome or *BRCA* mutation status.	Patients were randomly assigned in a 2:1 ratio to receive Olaparib tablets (300 mg twice daily) or PL for up to 24 months; all the patients received Bev at a dose of 15 mg/kg q3w for up to 15 months in total.	PFS.	Initial results indicate a PFS benefit when Bev + Olaparib (PARP inhibitor) combination therapy is used as maintenance. The median PFS was 22.1 months with Olaparib and 16.6 months with PL (*p* < 0.001). The addition of maintenance Olaparib provided a significant PFS and OS benefit, substantial in patients with homologous-recombination deficiency tumors, including those without a *BRCA* mutation.
**Cervical cancer**					
GOG-240 [69]	Phase III, multicenter, open label, randomized	452 patients with recurrent/persistent cervical cancer, not amenable to curative treatment with surgery and/or radiation therapy.	Arm I: paclitaxel + cisplatin/topotecan Arm II: paclitaxel + cisplatin/topotecan and Bev.	Risk of death. Risk of disease progression.	23% reduction in the risk of death (*p* = 0.007) in the Bev arm. 33% reduction in the risk of disease progression (*p* = 0.002) in the Bev arm. Adding Bev determined increased OS (17.0 vs. 13.3 months, *p* = 0.004) and RR (48% vs. 36%, *p* = 0.008). Bev was associated with an increased incidence of hypertension, thromboembolic events and gastrointestinal fistulas.
**Brain tumors**					
AVF3708g [70]	Phase II, open-label, non-comparative, randomized	167 patients with relapsed or progressive glioblastomas.	Arm I: Bev monotherapy Arm II: Bev + irinotecan	RR, PFS.	Improvement in PFS compared to historical controls: by 4.2 months when Bev is used as monotherapy and by 5.6 months when Bev was combined with irinotecan.
AvaGlio [71]	Phase III, first-line	Patients with newly diagnosed glioblastoma in first or second relapse.	Arm I: radiotherapy and temozolomide + PL. Arm II: radiotherapy and temozolomide + Bev.		Reduction in the risk of disease progression through the addition of Bev to radiotherapy + temozolomide, compared to control.

* The study included a third arm on which enrollment was discontinued early after the safety of the IFL + Bev regimen was assessed and considered acceptable. Abbreviations used in table: mCRC—metastatic colorectal cancer, IFL—standard chemotherapy containing 5-Fluorouracil, Leucovorin and Irinotecan; PL—placebo; Bev—Bevacizumab; q2w—every 2 weeks; q3w—every 3 weeks; FL—chemotherapy regimen containing 5-Fluorouracil and Leucovorin; OS—overall survival; PFS—progression free survival; RR—response rate; QoL—quality of life; HR—hazard ratio, ECOG—Eastern Cooperative oncology Group; NSCLC—non-small cell lung cancer; EGFR—epithelial growth factor receptor; AUC—area under the curve.

The pivotal AVF2107g study was a Phase III, multicenter, active-controlled study, sponsored by Genentech, that evaluated the efficacy and safety of Bevacizumab in combination with standard IFL chemotherapy (5-FU + Leucovorin ± Irinotecan) in human subjects with metastatic colorectal cancer [49,50]. The study enrolled 923 patients in 163 centers, who were randomized into three arms: 411 subjects—Placebo arm (patients received standard IFL chemotherapy and Placebo); 402 subjects—Bevacizumab arm (standard IFL and Bevacizumab 5 mg/kgc every 2 weeks); and 110 subjects—combination therapy arm but without irinotecan (FL chemotherapy: 5-FU + Leucovorin and Bevacizumab 5 mg/kgc every 2 weeks). The primary endpoint of the study was overall survival (OS), and secondary endpoints included progression-free survival (PFS), response rate, duration of response, quality of life, and patient safety. During the study, enrollment in the combination FL + Bevacizumab arm was discontinued due to safety concerns [51].

The AVF2107g study found that the addition of Bevacizumab to IFL chemotherapy improved OS compared with standard chemotherapy (20.3 months vs. 15.6 months, *p* < 0.0001) [49]. It also significantly improved progression-free survival during first-line treatment (*p* < 0.0001) [49,50,72]. The response rate to the treatment was 34.6% in the IFL + Placebo arm and 44.5% in the IFL + Bevacizumab arm, and the duration of response was 7.06 months and 10.35 months, respectively. The IFL + Bevacizumab combination therapy did not contribute to a more rapid deterioration of quality of life compared with standard first-line chemotherapy. These results were significant. The median time to deterioration was 2.73 months in the Placebo arm and 2.89 months in the Bevacizumab arm.

The supporting study AVF0780g [52] was a phase II, randomized, multicenter, multidose study sponsored by Genentech that evaluated the efficacy and safety of the use of recombinant human antibody Bevacizumab in combination with FL-type chemotherapy (5-FU + Leucovorin) in human subjects with metastatic colorectal cancer, as well as pharmacokinetic elements—determining the optimal dose for use in phase III studies. The study enrolled 104 patients with metastatic colorectal cancer who were randomized into three arms: FL chemotherapy, FL chemotherapy + Bevacizumab 5 mg/kgc, and FL chemotherapy + Bevacizumab 10 mg/kgc. The primary end-points were PFS and response rate. Both progression and response to therapy were assessed by an independent radiology clinic. Where the assessment could not be performed independently, the investigators completed these data (this occurred in the case of three patients in the LV + Bevacizumab 5 mg/kgc arm and 1 patient in the LV + Bevacizumab 10 mg/kgc arm). Disease progression on the treatment was observed in 26 of the 36 patients in the control group, in 22 of 35 patients treated with 5 mg/kgc Bevacizumab, and in 23 of 33 patients treated with 10 mg/kgc Bevacizumab. The median PFS expressed in months in the three groups were 5.2, 9 (*p* = 0.005), and 7.2, respectively (*p* = 0.236). A statistically significant improvement in PFS was observed for the 5 mg/kgc dose compared to the control group (statistical significance is maintained even after a Bonferroni adjustment), and the absence of a significant benefit for the higher dose of Bevacizumab. The study also evaluated overall survival in the 3 arms: 13.6 months (control arm), 17.7 months (5 mg/kgc arm, *p* = 0.52), and 15.2 months (10 mg/kgc arm, *p* = 0.978), respectively. No significant differences in OS were observed between the arms, but only a trend towards improvement in the 5 mg/kgc Bevacizumab arm [51].

After the approval of Bevacizumab, subsequent trials demonstrated the benefit of adding Bevacizumab to various forms of standard first-line chemotherapy (5-FU/leucovorin or XELOX)—trials AVF0780g and NO16966), or second-line chemotherapy (5-FU/leucovorin or FOLFOX)—E3200 trial, as well as the persistent benefit of Bevacizumab treatment in multiple lines (trial ML18147) [53,54,73,74].

In the decades since the introduction of Bevacizumab, more and more anti-angiogenic therapies have become available together with other targeted therapies targeting EGFR, kras, nras, and BRAF (for wild-type metastatic disease), or BRAF and HER2 (for metastatic colorectal cancer exhibiting these types of mutations). Also, new treatment approaches for metastatic colorectal cancer with microsatellite instability and mismatch repair deficiency based on immune checkpoint inhibitors have become available. Aflibercept, for instance, is a fusion protein targeting VEGF-A/B, and Pl-GF, may exhibit a stronger effect on inhibiting angiogenesis compared to Bevacizumab or Ramucirumab, due to multiple targeted pathways [75]. This effect seems to be consistent with trial results (e.g., VELOUR [76] which proved an improved median OS and median PFS in the FOLFIRI/aflibercept group versus control). The RR was also improved through the addition of Aflibercept. Given these results, it is no surprise that the European Society for Medical oncology ibercept combined with FOLFIRI can be an option for patients with DR or progression after oxaliplatin-containing treatment. Meanwhile, ESMO guidelines explicitly recommend Aflibercept as an alternative second-line treatment agent for RAS WT and RAS mutant patients [75]. Similar, Ramucirumab (an anti-VEGFR2 humanized monoclonal antibody), has gained FDA approval as a second-line treatment for metastatic colorectal cancer in combination with FOLFIRI type chemotherapy based on RAISE trial results proving improved OS and adequate safety profile [77]. Fruquintinib, a new-comer among anti angiogenetic drug, is a highly selective, long-term small molecule which selectively inhibits VEGFR, which has showed promising results in trials leading to it being recommended in some countries as a third-line agent for the treatment of patients with CRC [78]. Other antiangiogenetic drugs include multitargeting kinase inhibitors (TKIs) [79]. Regorafenib, one of these TKIs, was originally developed as an RAF1 inhibitor, but proved to block both VEGFR and Tie2 and to have an antiangiogenic effect. Moreover, this agent plays a role in regulating vessel stability and enhancing anti-tumoral immunity via macrophage modulation [80]. Regorafenib has been suggested as a third-line treatment for advanced CRC [81]. One of the most interesting particularities of Regorafenib is that, due to the fact that it is a TKI of the VEGF signaling pathways, it may have and therapeutic effect even in tumors resistant to VEGF inhibitors, and it might be an optimal combination partner for immune checkpoint inhibitors [82]. Aside from agents that block VEGF/VEGFR pathways, in CRC, another therapeutic target resulting in an antiangiogenetic effect is EGFR (e.g., Cetuximab and Panitumumab). Both these agents are FDA-approved as first-line treatment for CRC, but are not recommended as second or third line due to lack of benefits in PFS or OS in these settings [83]. Cetuximab exhibits an important immunogenic effect being a murine monoclonal antibody. Another concern about using EGFR inhibitors is the fact that in patients with CRC, the presence of kras mutations seems to be negatively corelated to response to anti-EGFR therapy and only patients with wild-type ras tumors receive a clinical benefit from anti-EGFR antibody therapy [84]. Some studies showed that using anti-EGFRs in the first-line setting for the right CRC should be avoided when other therapeutic alternatives are available [85] while for wild-type ras mCRC left-sided tumors, chemotherapy plus anti-EGFR drugs is the combination of choice as first-line treatment [86].

Regardless of these new therapies, Bevacizumab continues to play a role in the “standard-of-care” in patients with metastatic colorectal cancer, being recommended in combination with chemotherapy, both in initial, ulterior, and maintenance lines of therapy [87,88].

### 4.2. Lung Cancer

In 2006, the FDA approved Bevacizumab for first-line treatment of advanced non-small cell lung cancer (NSCLC), in combination with standard chemotherapy carboplatin/paclitaxel.

The approval came as a result of the pivotal study E4599, conducted under the aegis of the Eastern Cooperative Oncology Group (ECOG), between July 2001 and April 2004, which enrolled 878 patients with advanced, recurrent, and metastatic NSCLC (stage IIIB or IV). Patients were randomized to arm 1 (standard chemotherapy—444 subjects) and arm 2 (carboplatin + paclitaxel in combination with Bevacizumab—434 subjects) [89]. The endpoint of this study was overall survival (OS), with reported results being 10.3 months in arm 1 versus 12.3 months in arm 2 (hazard ratio for death = 0.79; *p* = 0.003). The PFS values (secondary endpoint) were 4.5 months (arm 1) and 6.2 months (arm 2), respectively—the hazard ratio for progressive disease being 0.66, *p* < 0.001—and the corresponding response rates to therapy were 15% and 35%, respectively (*p* < 0.001). There were 15 deaths associated with Bevacizumab therapy, including 5 due to massive pulmonary hemorrhage. The study concluded that the addition of Bevacizumab to standard chemotherapy (carboplatin + paclitaxel) in the first-line treatment of patients with advanced, recurrent, or metastatic NSCLC improved overall survival, but at the risk of more frequent and severe adverse events. After the study was completed, a planned analysis of overall survival by tumor histology was performed, which showed that overall survival in adenocarcinoma patients was twice as high compared to the total patient population [55].

The AVAiL study (BO17704) [90] is another European, international, phase III, placebo-controlled clinical trial that enrolled 1043 patients with advanced or recurrent NSCLC, subsequently randomized into three arms—arm one which included 326 subjects who received standard chemotherapy (cisplatin + gemcitabine) + placebo, arm two with 331 subjects who received standard chemotherapy + 7.5 mg/kg Bevacizumab, and arm three with 329 subjects who received standard chemotherapy + 15 mg/kg Bevacizumab. Bevacizumab or Placebo were administered until disease progression. The final analysis confirmed the efficacy of adding Bevacizumab to the cisplatin + gemcitabine combination chemotherapy, demonstrating a statistically significant PFS benefit in both the 7.5 and 15 mg/kg groups (*p* = 0.0003 and *p* = 0.0456, respectively). The PFS benefit did not translate into overall survival benefits, with OS being >13 months in all arms (probably due to the efficacy of second-line therapies that were administered to patients after progression [91]. Similar effects were also observed when only patients over 65 years of age were included in the analysis. In these patients, there was no change in the safety profile compared to the entire group [92] thus proving the safety of Bevacizumab in older patients.

Subsequent studies also confirmed the improvement in PFS in the case of combination therapy (chemotherapy + Bevacizumab), but the results in terms of overall survival were variable, both in first-line therapy and in subsequent lines or maintenance [56,89,90,91,92,93]. In the AVAPERL study—MO22089—the combination therapy Bevacizumab/permetrex proved superior in maintenance after achieving disease stability with cisplatin/permetrex chemotherapy + Bevacizumab, resulting in a 50% reduction in the risk of NSCLC progression compared with bevacizumab monotherapy (median PFS: 10.2 vs. 6.6 months), but without an overall survival benefit [57,58].

In 10–20% of NSCLC cases, certain carcinogenic mutations (the most common being EGFR aberrations) are present and can be targeted (patients with EGFR mutations are usually treated with tyrosine kinase inhibitors in monotherapy, such as erlotinib [94,95]). Although several tyrosine kinase inhibitors therapies are available, resistance eventually develops in almost all patients. For this reason, the JO25567 study was initiated, which compared erlotinib + Bevacizumab with erlotinib alone, demonstrating the superiority of the combination therapy (46% reduction in the risk of progression; hazard ratio = 0.54; *p* = 0.015) [94,96]. Based on this study, the erlotinib + Bevacizumab combination therapy was approved for the treatment of patients with advanced/metastatic NSCLC with EGFR mutations. The results of the JO25567 study were also confirmed by the results of two phase 3 studies (the NEJ026 and Artemis studies) [61,94,97]. In addition to EGFR mutations, patients with NSCLC may harbor other molecular/genetic aberrations that can be targeted by targeted therapies, such as neurotrophic tropomyosin receptor kinase (NTRK), anaplastic lymphoma kinase (ALK), BRAF, and ROS1 [97,98]. Furthermore, the introduction of immune checkpoint inhibitors has changed the treatment landscape of NSCLC, with some of these drugs achieving durable responses in different subsets of patients. The combined effect of immune checkpoint inhibitors with Bevacizumab has been the focus of numerous studies. For example, the IMpower150 trial can be mentioned; this trial observed a 38% reduction in the risk of disease progression (*p* < 0.001) when patients were treated with Bevacizumab and chemotherapy combined with an immune checkpoint inhibitor (Atezolizumab) rather than just Bevacizumab + chemotherapy. These benefits which were observed regardless of EGFR and ALK status, led to the approval of the combination in the first-line treatment of NSCLC [99]. Although the NSCLC treatment landscape has evolved substantially in recent decades, Bevacizumab remains an important part of treatment as a single agent, in combination with systemic chemotherapy, or in combination with other targeted therapies such as erlotinib and atezolizumab.

### 4.3. Breast Cancer

Bevacizumab was approved by the FDA for use in patients with metastatic breast cancer in February 2008 under the accelerated approval program.

The accelerated approval of Bevacizumab was based on the E2100 study—a randomized, open-label trial organized by the NCI (National Cancer Institute) and ECOG (Eastern Cooperative Oncology Group), which enrolled 722 patients with locally advanced, recurrent, or metastatic breast cancer who had not previously received Bevacizumab. Patients were randomized (1:1) to receive paclitaxel alone or paclitaxel + Bevacizumab [100]. The primary endpoint was progression-free survival (PFS). The addition of Bevacizumab to chemotherapy resulted in a 5.5-month increase in median progression-free survival (hazard ratio = 0.48; 95% CI 0.39–0.61; *p* < 0.0001), but no statistically significant improvement in OS was observed (hazard ratio = 0.87; 95% CI 0.72–1.05; *p* = 0.137). The tumor response rate was higher in the Bevacizumab arm compared with the control arm (48.9% vs. 22.2%) [101].

### 4.4. Renal Cell Carcinoma

Patients with renal cell carcinoma (RCC) often present in advanced or metastatic stage, resulting in a very poor 5-year survival rate and a median overall survival of approximately 7 months [102]. Prior to the development of targeted therapies, the standard of care for these patients was surgery and INF-α [103,104]. Bevacizumab was the first angiogenesis inhibitor to be approved for use in first-line therapy of renal cell carcinoma, after the pivotal AVOREN trial proved a 37% reduction in the risk of disease progression (hazard ratio = 0.63; *p* = 0.0001) with the addition of Bevacizumab to INF-α therapy compared with INF-α alone [103,105,106], which, however, did not translate into an overall survival benefit. The results of the pivotal study were also confirmed by the CALGB 902065 study [107,108].

Tumor angiogenesis is a particular feature in renal cell carcinoma and, over time, numerous anti-angiogenic agents have been approved for this pathology: VEGF/VEGFR pathway inhibitors (tivozanib), multi-kinase inhibitors (sorafenib, pazopanib, sunitinib, axitinib, lenvatinib, cabozantinib), or mTOR inhibitors (temsirolimus, everolimus). Bevacizumab remains, even under these conditions, an important therapeutic option, and there are studies (such as the IMmotion trial151) which investigate the efficacy of combination therapy consisting of Bevacizumab and other classes of immune modulators/targeted therapies (such as atezolizumab [109], erlotinib [110], or nivolumab [111]), with encouraging results.

### 4.5. Ovarian, Fallopian Tube, and Primary Peritoneal Cancer

These entities are usually studied together due to their common characteristics both in terms of evolution and treatment characteristics. They are aggressive cancers, with insidious evolution, with a tendency towards extensive intra-abdominal dissemination rather than hematogenous metastasis, and present in most cases in advanced stages. Before the advent of targeted therapies, the only therapeutic options were cytoreductive surgery and platinum-based chemotherapy [112,113,114], under which most cases achieved initial remission of the disease only to later relapse (with a particularly high recurrence rate). Bevacizumab was the first targeted therapy approved for the treatment of advanced primary ovarian, fallopian tube, and peritoneal cancers.

Its approval as a first-line treatment was granted following the publication of the results of the pivotal GOG-0218 trial—a phase III, double-blind, placebo-controlled trial. The study enrolled 1873 women with stage III or IV primary ovarian, fallopian tube, or peritoneal cancer who had undergone suboptimal cytoreductive surgery which were randomized 1:1:1 to platinum-based chemotherapy, platinum-based concomitant therapy + Bevacizumab 15 mg/kg, and platinum-based concomitant therapy + Bevacizumab followed by Bevacizumab maintenance. The final results of the study showed that the addition of Bevacizumab to chemotherapy resulted in a statistically significant increase in PFS (from 10.3 to 14.1 months—hazard ratio 0.717; CI95% 0.625–0.824; *p* ≤ 0.001) [115]. Although there was no significant difference in overall survival between the arms, in the subgroup of patients with stage IV disease, combined therapy followed by Bev maintenance led to an improvement in OS (median OS 42.8 vs. 32.6 months, hazard ratio 0.75).

The efficacy of Bevacizumab in the first-line treatment of patients with advanced/recurrent ovarian, tubal, or peritoneal cancer has been supported by the results of subsequent studies (ICON7, OCEANS, GOG-0213, AURELIA) [66,116,117,118,119,120].

The ICON7 study was a phase III, open-label trial that enrolled 1528 women diagnosed with stage III or IV primary ovarian, fallopian tube, or peritoneal cancer who underwent cytoreductive surgery (including, unlike GOG-0218, both sub-optimally operated patients and patients without visible signs of disease), who were subsequently randomized 1:1 to receive carboplatin + paclitaxel or combination therapy of carboplatin + paclitaxel and Bevacizumab 7.5 mg/kg. The final published results did not demonstrate a significant benefit in PFS or OS. Only the analysis of the subgroup of high-risk patients demonstrated an improvement in PFS in the Bevacizumab-treated arm (16.0 months vs. 10.5 months; hazard ratio 0.73; CI95% 0.61–0.88; *p* = 0.001) [120]. The published conclusions that there are sufficiently strong data to support the use of Bevacizumab based on the stratification of patients according to risk class and tumor burden were, however, publicly contested [121] on the following grounds: 1. the proven benefit is based on a subgroup analysis and the trial is open-label (which confers lower statistical power); 2. similar benefits to those proven can also be obtained by using Bevacizumab after disease progression (without the need for use from the beginning of treatment, the existing clinical evidence supporting this fact with equal strength); 3. superior PFS but also OS benefits were obtained in the JGOG3016 trial using dose-dense paclitaxel as monotherapy in patients with residual disease ≥ 1 cm (whom the ICON7 study classified as high-risk patients); 4. the increased costs of a therapy that has not been proven clinical efficacy and high toxicity. In light of these objections, the authors of the original study continued to support the beneficial effect of Bevacizumab in high-risk patients but admitted that the optimal time to initiate therapy (first-line or after disease progression) is still unclear [122].

However, years later, Bevacizumab remains an important standard of care and the only anti-angiogenic agent approved for the treatment of advanced/recurrent primary ovarian, fallopian tube, and peritoneal cancers. Recently, new therapeutic options have begun to emerge that appear to bring significant clinical benefits, such as PARP inhibitors, which target tumors with BRCA mutations or other DNA repair deficiencies. Several phase 3 trials (currently ongoing or recently completed) are investigating the benefits of Bevacizumab in combination with PARP inhibitors (PAOLA-1 trial) or with immune checkpoint inhibitors (IMagyn050, ATALANTE, AGO OVAR 2.29, and NRG-GY009 trials) [123,124,125,126,127]. The initial results from PAOLA-1 indicate a progression-free survival benefit when Bevacizumab + PARP inhibitor Olaparib combination therapy is used as maintenance [67].

### 4.6. Cervical Cancer

Patients with persistent/recurrent cervical cancer have a limited survival of 1–2 years in the context of available treatment (chemotherapy) with only short-term responses [128]. Bevacizumab was the first targeted therapy approved for this condition, which brought significant progress, filling a large unmet medical need and establishing the standard of care for this subset of patients [129,130].

The pivotal GOG-0240 trial is a phase III, multicenter, open-label, randomized trial that enrolled 452 patients with recurrent/persistent cervical cancer and is the trial that led to the approval of Bevacizumab for recurrent/persistent cervical cancer t. The trial demonstrated a 23% reduction in the risk of death (hazard ratio 0.77; *p* = 0.007) and a 33% reduction in the risk of disease progression (hazard ratio 0.67; *p* = 0.002) by adding Bevacizumab to paclitaxel + cisplatin/topotecan compared with chemotherapy alone [69,131].

Recently, Pembrolizumab and Atezolizumab (immune checkpoint inhibitors) have been approved for use in recurrent, progressive, or metastatic cervical cancer expressing PD-1 (programmed cell death protein 1) [132,133]. There are current phase III studies (such as BEATcc) investigating the efficacy of adding an immune check point inhibitor (Atezolizumab) to Bevacizumab and chemotherapy in patients with persistent or recurrent cervical cancer [134,135].

### 4.7. Brain Tumors

Bevacizumab was approved by the FDA for the treatment of glioblastomas, rare but highly aggressive tumors, with a median survival of approximately 15 months despite aggressive chemo-radiotherapy. Treatment options are limited to drug agents that can cross the blood–brain barrier, but this ability also determines a high potential for adverse reactions.

The pivotal study, AVF3708g, that was the basis for the approval of Bevacizumab in the treatment of relapsed or progressive glioblastomas is a phase II, open-label, non-comparative study that enrolled 167 subjects who were subsequently randomized to receive monotherapy with Bevacizumab or Bevacizumab + irinotecan. The study demonstrated an improvement in progression-free survival by 4.2 months when used alone and by 5.6 months when Bevacizumab was combined with irinotecan compared to historical controls [136]. Based on these results, Bevacizumab was approved in the United States for the treatment of relapsed or progressive glioblastoma. This indication has not been approved in Europe.

The AvaGlio study (BO21990), a phase III study that investigated the utility of using Bevacizumab in the first line in patients with glioblastoma, demonstrated a reduction in the risk of disease progression through the addition of Bevacizumab to the combination therapy (radiotherapy + temozolomide), compared to the combination therapy alone. Similar results were obtained in the RTOG 0825 study [137,138]. As in the other studies conducted for other indications, none of the three studies above showed a correlation between the observed PFS benefits and an overall survival benefit. However, there is epidemiological data that provides indirect evidence supporting that Bevacizumab has a positive impact on overall survival (data from the United States cancer registry showed an increase in median survival in patients with glioblastoma coinciding with the approval of Bevacizumab for this indication [139]).

Although it does not provide an overall survival benefit, Bevacizumab was the first pharmacological agent used in the treatment of patients with glioblastoma leading to a longer maintenance of optimal quality of life and performance status. Furthermore, Bevacizumab treatment has been associated with a decrease in the need for glucocorticoids, which are used to treat peritumoral cerebral edema, but which can cause potentially serious side effects, causing notable morbidity [137].

## 5. Controversies Regarding Bevacizumab

### 5.1. Approval Process and Pivot-Trials Biases

Over the years, a number of controversies have focused on Bevacizumab, culminating with the withdrawal of the FDA approval for use in breast cancer patients. The FDA grants accelerated approval for drugs that treat severe conditions for which there are currently no effective treatments based on surrogate endpoints for overall survival that can be obtained and evaluated after a shorter period of time (such as clinical response or progression free survival) and which suggests an important clinical benefit to patients. However, the FDA requires the manufacturer of a drug approved by accelerated procedure to present the mature overall survival results after the completion of the trials [101], this being the manufacturer’s responsibility to prove these benefits.

As a condition of the accelerated approval for Bevacizumab in breast cancer patients, the FDA required the manufacturer to submit the final results of two randomized, placebo-controlled trials that were currently underway (AVADO and RIBBON 1) to verify the effect of Bevacizumab on PFS demonstrated by the pivotal study (E2100) and provide additional data on its effect on overall survival.

The AVADO study (BO17708) was a double-blind, placebo-controlled trial that enrolled 736 patients with Her-2-negative metastatic breast cancer who had not received prior chemotherapy, with three arms: docetaxel + placebo, docetaxel + Bevacizumab 7.5 mg/kg, and docetaxel + Bevacizumab 15 mg/kg. The addition of Bevacizumab 7.5 mg/kg to systemic therapy resulted in a 30% increase in PFS (hazard ratio = 0.70; 95% CI: 0.55–0.90), but with an absolute difference in PFS of only 0.8 months, while the addition of Bevacizumab 15 mg/kg resulted in a 39% increase in PFS (hazard ratio = 0.62; 95% CI: 0.48–0.79) with an absolute difference of 0.88 months. Objective responses were observed in 44% of patients in the docetaxel + placebo arm, 55% in the docetaxel + Bevacizumab 7.5 mg/kg arm (*p* value 0.0295) and 63% in the docetaxel + Bevacizumab 15 mg/kg arm (*p* value 0.0001). Mature survival data showed an absolute decrease in survival in both arms of the AVADO study to which Bevacizumab was added, although these differences were not statistically significant. There was also a significant increase in NCI-CTCAE grade 3–5 adverse events and the need for treatment discontinuation [140]. The addition of bevacizumab resulted in a 20.2% increase in the rate of NCI-CTCAE (NCI Common Toxicity Criteria for Adverse Effects) grade 3–5 complications and a 1.7% increase in therapy related mortality [101].

The double-blind, parallel-groups, RIBBON 1 trial also known as BO20094, enrolled 1237 women with Her-2-negative recurrent or metastatic breast cancer chemotherapy naive, later randomized 2:1 to receive either chemotherapy (anthracycline/taxanes—622 patients or capecitabine—615 patients) in combination with either bevacizumab or placebo. The addition of Bevacizumab to anthracycline/taxanes chemotherapy resulted in a 36% increase in progression free survival (HR 0.64; 95% CI 0.52–0.80), but with an absolute difference in PFS of only 1.2 months. The response rate to therapy was higher in the Bevacizumab arm, with an absolute increase of 13.5%. The analysis of mature survival favored the placebo arm (HR 1.11; 95% CI 0.86–1.43). The addition of Bevacizumab to capecitabine resulted in similar results with a 31% increase in PFS (hazard ratio = 0.69; 95% CI 0.56–0.84), an absolute difference in only 1.2 months. PFS was 2.9 months, with a response rate of 11.8% higher in the Bevacizumab arm. However, in the capecitabine cohort, an overall survival benefit was observed in favor of the Bevacizumab arm (hazard ratio = 0.88; 95% CI 0.69–1.13) [141]. In both cohorts, the incidence of NCI-CTCAE grade 3–5 adverse events was approximately twice as high in the Bevacizumab arms compared with the placebo arms [141].

The magnitude of the improvement in PFS observed in the AVADO and RIBBON 1 trials failed to confirm the clinical benefits suggested by the E2100 trial, which was the basis for the accelerated approval of the drug. The magnitude of the treatment effect is clinically important because it allows the assessment of benefit in relation to drug toxicity. The addition of bevacizumab to chemotherapy resulted in an increased rate of serious adverse events and adverse events attributable to the study drug in both the AVADO and RIBBON1 trials. Mature overall survival data favored the placebo arms in both the AVADO and the anthracycline/taxanes cohorts of the RIBBON 1 trial. In light of the above, the FDA initiated an accelerated approval withdrawal procedure and in November 2011 announced the withdrawal of approval for the use of Bevacizumab as a first-line treatment, in combination with paclitaxel, for advanced/recurrent or metastatic Her-2-negative breast cancer [142,143,144]. In Europe, the European Medicines Agency maintained its approval for this indication despite the data from the two studies, issuing the following statement: “For Avastin in combination with paclitaxel, the Committee concluded that the benefits continue to outweigh the risks, as the available data convincingly showed that it prolongs progression-free survival in patients with breast cancer, without a negative effect on overall survival” [145]. The reasons for the different decisions of the two regulatory agencies based on the same clinical evidence are unclear. However, a lack of transparency in the EMA decisions should be noted, which should be contrasted with the publicly available reviews and discussions of the FDA on Bevacizumab.

Also, the pivotal studies upon which Bevacizumab was approved for colorectal cancer (AVF2107g and AVF0780g) had many methodological shortcomings related mainly to patient eligibility, treatment protocol, and data collection deficiencies. In AVFG2107g, 39.9% of patients in the control arm and 49.5% of patients in the study arm experienced major protocol violations. Taking this into account, the FDA demanded that the overall survival analysis to be redone after excluding patients with major protocol violations, but the OS benefit remained significant (*p* = 0.0012) [49]. The supporting study, AVF0780g, also faced similar shortcomings. It should be noted that three patients in the control group and six in the intervention groups discontinued treatment before the first scheduled progression assessment. Premature discontinuation of the intervention, especially in small-sample studies such as this one, may affect the results obtained or the power of the statistical tests applied. Seven of these nine patients were censored and excluded from the progression analysis. It should be noted that after multiplicity adjustments, the statistical significance of the difference between the control and 5 mg/kg arms is lost [51].

### 5.2. Resistance to Bevacizumab Therapy

Another concern about all anti-angiogenetic drugs (including Bevacizumab) is a lack of enduring clinical response which limit the initial success of such therapies. Although, the vast majority of tumors are intrinsically responsive to angiogenesis inhibitors, they rapidly adapt and escape the effect of the drugs, and this resistance to therapy is a practical limitation of usage and often translates into an overall survival that is not prolonged. Bevacizumab therapy may paradoxically lead to the selection of cellular clones (including Cancer Stem-like Cells) with a selective advantage of survival in hypoxic conditions, especially in the necrotic center of the tumor where the drug does not fully penetrate. These cells can fuel tumor self-renewal and are usually more aggressive. Animal models have shown that treatment with VEGF-inhibitors increases the invasiveness of the primary tumors and enhances metastasis to liver and lymph nodes [146]. Moreover, the inhibition of VEGF/VEGFR axis can cause compensatory reactions and release of alternative stimulatory factors [147,148,149,150,151] and recruitment of other pro-inflammatory/pro-angiogenic cells [152]. These resistance mechanisms severely limit the effectiveness of Bevacizumab as a single-agent therapy. Also, there are currently no adequate biomarkers to predict the resistance to therapy. Due to a high proportion of non-responders and patients which develop resistance to therapy, there is an unmet need for novel strategies to compensate for these limitations of the antiangiogenetic therapies. This issue is currently addressed by numerous in-progress clinical trials studying the effect of Bevacizumab in combination with other immunomodulatory and targeted drugs, many of which are showing promising initial results.

## 6. Conclusions

In conclusion, Bevacizumab, one of the first drugs targeting tumoral microenvironment, is an essential oncologic agent blocking the VEGF/VEGFR pathway of angiogenesis. Despite a history of more than 20 years (since its first approval for patients with metastatic colorectal cancer) marked by success but also numerous controversies (ranging from methodological errors of clinical trials to withdrawal of approval for human usage in breast cancer patients, from discussions about severe side effects to resistance to therapy and limited efficacity), Bevacizumab continues to provide an optimal therapeutic option for many solid tumors that previously had little or no means of treatment, improving otherwise bleak outcomes. Even after the development of other targeted agents, Bevacizumab continues to remain a key element of many therapeutic regimens both as monotherapy and in combination with these newer drugs.

## Data Availability

No new data was created for this article. All source materials are available online and are cited in the references section.
